# Ocean Acidification Accelerates the Growth of Two Bloom-Forming Macroalgae

**DOI:** 10.1371/journal.pone.0155152

**Published:** 2016-05-13

**Authors:** Craig S. Young, Christopher J. Gobler

**Affiliations:** Stony Brook University, School of Marine and Atmospheric Sciences, Southampton, New York, United States of America; University of Hong Kong, HONG KONG

## Abstract

While there is growing interest in understanding how marine life will respond to future ocean acidification, many coastal ecosystems currently experience intense acidification in response to upwelling, eutrophication, or riverine discharge. Such acidification can be inhibitory to calcifying animals, but less is known regarding how non-calcifying macroalgae may respond to elevated CO_2_. Here, we report on experiments performed during summer through fall with North Atlantic populations of *Gracilaria* and *Ulva* that were grown in situ within a mesotrophic estuary (Shinnecock Bay, NY, USA) or exposed to normal and elevated, but environmentally realistic, levels of pCO_2_ and/or nutrients (nitrogen and phosphorus). In nearly all experiments, the growth rates of *Gracilaria* were significantly increased by an average of 70% beyond in situ and control conditions when exposed to elevated levels of pCO_2_ (*p*<0.05), but were unaffected by nutrient enrichment. In contrast, the growth response of *Ulva* was more complex as this alga experienced significantly (*p*<0.05) increased growth rates in response to both elevated pCO_2_ and elevated nutrients and, in two cases, pCO_2_ and nutrients interacted to provide a synergistically enhanced growth rate for *Ulva*. Across all experiments, elevated pCO_2_ significantly increased *Ulva* growth rates by 30% (*p*<0.05), while the response to nutrients was smaller (*p*>0.05). The δ^13^C content of both *Gracilaria* and *Ulva* decreased two-to-three fold when grown under elevated pCO_2_ (*p*<0.001) and mixing models demonstrated these macroalgae experienced a physiological shift from near exclusive use of HCO_3_^-^ to primarily CO_2_ use when exposed to elevated pCO_2_. This shift in carbon use coupled with significantly increased growth in response to elevated pCO_2_ suggests that photosynthesis of these algae was limited by their inorganic carbon supply. Given that eutrophication can yield elevated levels of pCO_2_, this study suggests that the overgrowth of macroalgae in eutrophic estuaries can be directly promoted by acidification, a process that will intensify in the coming decades.

## Introduction

Ocean acidification is changing the chemistry of the ocean. Beyond reducing pH, the anthropogenic delivery of CO_2_ into surface oceans this century will differentially effect various pools of inorganic carbon, with CO_2_ and HCO_3_^-^ expected to increase 260% and 20%, respectively, and CO_3_^2-^ levels expected to decrease 60% [1]. As the total dissolved inorganic carbon (DIC) pool shifts towards these predicted values, marine flora and fauna are expected to have a varied response with lower availability of CO_3_^2-^ inhibiting the growth of calcifying organisms [2–4] and higher CO_2_ levels potentially benefiting some, but not all, photosynthetic organisms [2, 5–6].

The extent to which uncalcified marine macroalgae benefit from anthropogenically-induced changes in carbonate chemistry is complex and not fully understood. While CO_2_ is an important carbon source for photosynthesis, the likelihood of elevated CO_2_ benefiting autotrophs is partly dependent on photosynthetic pathways utilized by algae. C_3_ plants that utilize RuBisCO as their initial carboxylating enzyme experience loss of fixed carbon due to photorespiration and may benefit from increased CO_2_ concentrations since RuBisCO is not substrate-saturated at current CO_2_ levels [1, 7]. In contrast, C_4_ plants that utilize phosphenolpyruvate carboxylase (PEPC) experience little photorespiratory loss due to use of carbon concentrating mechanisms (CCM) and thus may not benefit from increased CO_2_ since PEPC is substrate-saturated at current CO_2_ levels [1, 7]. Marine macroalgae acquire carbon through direct diffusive uptake of CO_2_ as well as active transport of CO_2_ and HCO_3_^-^ [8]. Although the majority of macroalgae are C_3_ plants, they often make use of CCMs and extracellular carbonic anhydrase (CA) to convert HCO_3_^-^ to CO_2_ for use by RuBisCO [1, 8–10]. However, there is significant variation in the photosynthetic strategies employed by different macroalgae regarding the use of extracellular CA as well as the degree to which HCO_3_^-^ and/or CO_2_ can or cannot be utilized for photosynthesis [8]. Macroalgal growth in response to elevated pCO_2_ can also be manifested through non-photosynthetic means. Webber et al. [11] and Roger et al. [12] found that acclimation to elevated CO_2_ can result in decreased concentrations of RuBisCO, but results in an increase in soluble carbohydrate content that could enhance growth rates and alter the total carbon content of algal tissues.

Beyond the progressively increasing levels of CO_2_ in the world’s oceans due to the combustion of fossil fuels, there are strong sources of CO_2_ in coastal zones [13]. One of the most prominent CO_2_ sources in coastal zones appears to be eutrophication-enhanced microbial respiration [14–16]. The accumulation of respiratory CO_2_ from the degradation of excessive organic matter can lower seawater pH and commonly result in CO_2_ levels (>1,000 μatm) not predicted to occur in open ocean regions for more than a century [16]. The combination of excessive nutrients and elevated CO_2_ could have a variety of impacts on primary producers. It has been well-established that with excessive nutrient loading, dominance among benthic autotrophs can shift from seagrasses to fast-growing, ephemeral macroalgae such as *Ulva* and *Gracilaria* [17–18]. Furthermore, some species of *Ulva* including *Ulva*. *rigida* and *U*. *lactuca* have been shown to experience increased growth under elevated CO_2_ concentrations [19–20], while others have not [21]. Additionally, elevated CO_2_ levels could aid in the assimilation of nutrients by *Ulva* [22]. *Ulva* is well-known for the formation of green tides along eutrophied coastlines such as Brittany, France, and Qingdao, China [23–25]. Common rhodophytes such as *Gracilaria* have been shown to bloom in response to high nutrient concentrations [26] and, like some *Ulva*, may also benefit from elevated CO_2_ concentrations, although this has never been examined. In general, the dynamics of macroalgal communities in response to eutrophication and elevated CO_2_ are difficult to generalize, as both the slow- and fast-growing species have been hypothesized to benefit from elevated CO_2_ and nutrients and studies assessing the response of macrophytes to elevated CO_2_ have been limited [1].

The objective of this study was to assess how elevated concentrations of CO_2_ alone, and combined with elevated nutrient levels, affect the growth rates of two common species of temperate, bloom-forming macroalgae; the rhodophyte, *Gracilaria*, and the chlorophyte, *Ulva*. The overabundance of these macroalgae is commonly interpreted as a symptom of eutrophication and their overgrowth within estuaries can have a series of negative impacts on marine plants and animals [18, 27–28]. Macroalgae were exposed to ambient and elevated concentrations of CO_2_ with and without nutrient enrichment during experiments performed throughout their growing season and their growth responses, δ^13^C signatures, and elemental composition were evaluated.

## Methods

### Macroalgae Collection and Preparation

**Fig 1:**
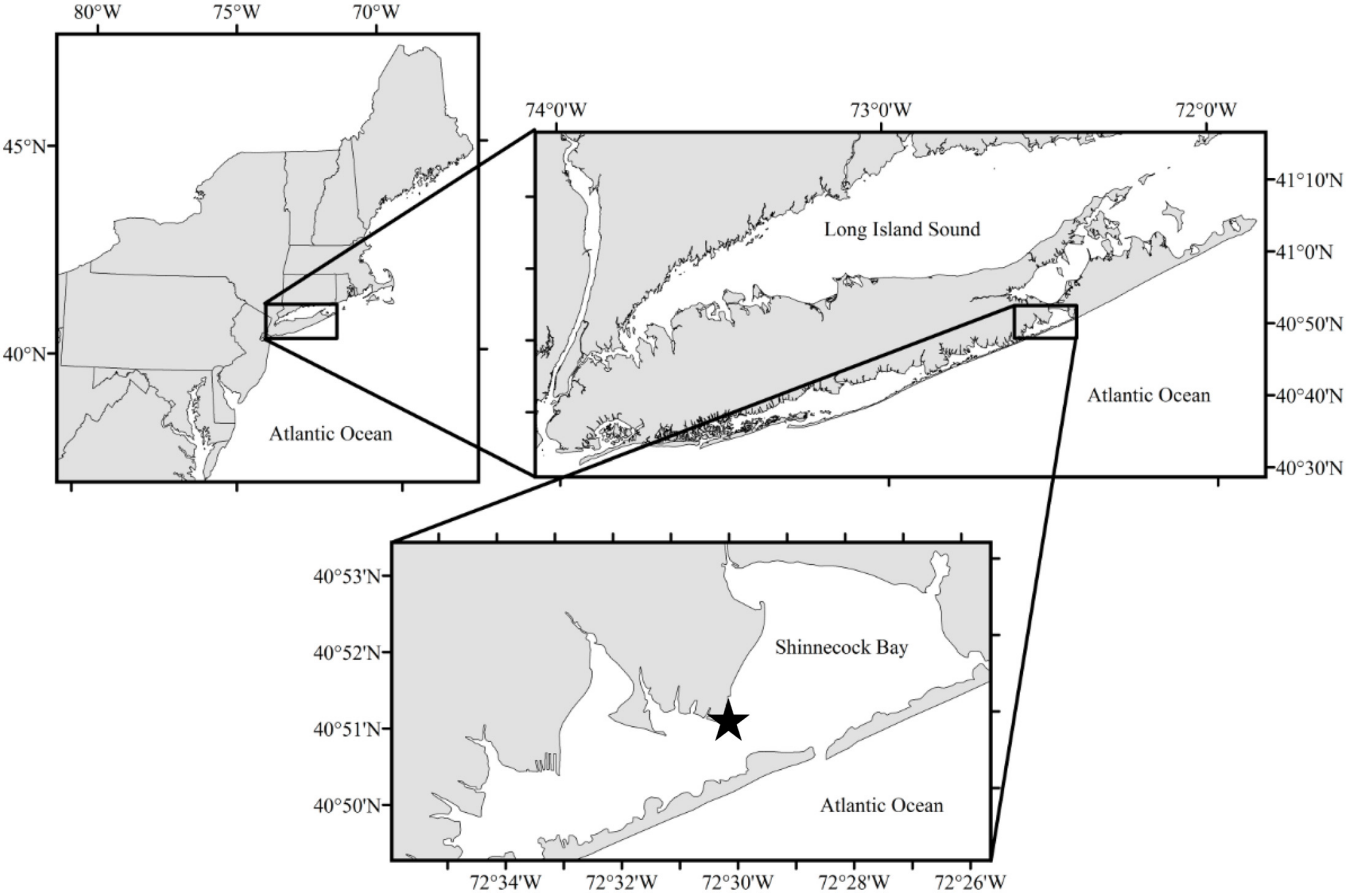
Shinnecock Bay, NY, USA. Map of Shinnecock Bay, NY, USA. The star represents the shallow-water region where macroalgal collections occurred and in situ experiments were performed.

Macroalgae used for this study were collected from shallow regions of eastern Shinnecock Bay, NY, USA (40.85° N, 72.50° W; Fig 1) during low tide. Permission to access the water and collect the water and macroalgae was received from the Southampton Town Trustees, Southampton, NY, USA, who hold jurisdiction over Shinnecock Bay. Collections targeted large, well-pigmented, robust fronds of *Ulva* and *Gracilaria* that were transferred to dark, temperature-controlled containers filled with seawater and returned to the Stony Brook Southampton Marine Science Center within 15 minutes of collection. Individual thalli of *Gracilaria* approximately 5 cm in length were cut from the main plant and spun in a salad spinner to remove debris and epiphytes. Samples were then extensively rinsed with filtered (0.2 μm) seawater and placed into the salad spinner a second time to further remove debris, epiphytes, and excess seawater. *Ulva* samples were prepared by use of a small brass ring to cut circular sections approximately 3 cm in diameter from a singular, large sheet of *Ulva* with care taken to avoid the outer, potentially reproductive region of the plant [29]. *Ulva* circles were brought through the same cleaning procedures described for *Gracilaria*. Five additional circular samples of *Ulva* were created from the same vegetative plant and were placed between two transparency films and frozen for future analysis described below. All samples were weighed on an A&D EJ300 digital scale (± 0.01 g) to obtain initial wet weights in grams. To prevent desiccation, all samples were kept in individual, 100 mL filtered (0.2 μm) seawater-filled containers after spinning prior to use in experiments.

### In situ growth experiments

In situ growth experiments with *Gracilaria* and *Ulva* were performed to assess the rate at which the macroalgae grew within the region of Shinnecock Bay from which they were collected. Experiments were performed monthly from June through November with two experiments performed September and October, for a total of eight experiments. Quadruplet, 0.25 m^2^ incubation cages constructed from ~1 cm^2^ wire mesh were attached to a four-armed (25 cm) umbrella fishing apparatus on a line with surface flotation and a bottom weight that kept cages suspended at 0.2 m [29]. Discrete and continuous measurements of light and temperature present during experiments were made using a LI-COR LI-1500 light sensor logger and HOBO pendant temperature and light loggers, respectively. Quadruplet thalli of each macroalgae species were placed in each cage for ~7 days after which the samples were recovered, brought to the lab, and rinsed, spun, re-rinsed, re-spun, and weighed as described above. *Gracilaria* samples were placed into small freezer bags for further analysis, whereas *Ulva* samples were placed between two transparency films and flattened with care to minimize folds. The surface areas of the experimental *Ulva* samples, in addition to the five initial *Ulva* samples were analyzed using SigmaScan Pro 5 [29]. Weight-based growth rates for both species were determined using the relative growth rate formula (growth d^-1^) = (ln W_final_−ln W_initial_) / (Δt) where W_final_ and W_initial_ are the final and initial weights in grams and Δt is the duration of the experiment in days.

### Assessing the effects of elevated nutrients and pCO_2_

Parallel experiments were established to assess the effects of elevated nutrients and pCO_2_ on the growth of *Gracilaria* and *Ulva*. Thirty-six, 2.5 L polycarbonate bottles were acid-washed (10% HCl), liberally rinsed with deionized water before use, and rinsed and filled with 0.2 μm filtered seawater from eastern Shinnecock Bay. Experimental bottles were placed in an environmental control chamber set to approximate the temperature (16–21°C) and light intensity (~450–500 μmol s^-1^ m^-2^ on a 14 h: 10 h light dark cycle) present during in situ experiments and were randomly assigned, in triplicate (n = 3), to one of four treatments for each species: a control with ambient levels of pCO_2_ (~400 μatm) and no nutrients added, a treatment of enhanced nutrient levels (50μM nitrate, 3 μM phosphate), a treatment of elevated pCO_2_ (~2000 μatm), and a treatment of elevated pCO_2_ and nutrient levels (~2000 μatm, 50μM nitrate, 3 μM phosphate). These nutrient and pCO_2_ levels were higher than levels present at the collection site, but consistent with concentrations present in eutrophic US East Coast estuaries [16, 29]. Each bottle was aerated via a 1 mL, polystyrene serological pipette inserted to the bottom of each experimental bottle and via tygon tubing to an air source. Bottles were subjected to the control level of pCO_2_ (~400 μatm) and elevated (~2000 μatm) via use of a gas proportionator system (Cole Parmer^®^ Flowmeter system, multitube frame) that mixed ambient air with 5% CO_2_ gas [3]. The δ^13^C of this tanked CO_2_ gas was determined to be -27.7‰ by syringe injection into a split/splitless inlet of a continuous flow gas chromatograph isotope ratio mass spectrometer (cf-GCIRMS, Finnegan MAT 253) using a 0.25μm x 30m poraplot column and a secondary standard referenced to V-PDB in the laboratory of Dr. John Mak (Stony Brook University). The mixtures of air and CO_2_ gas were delivered at a net flow rate of 500 ± 5 mL min^-1^ through an 18-way gang valve into the serological pipettes that fit through an opening drilled into the closed cap to the bottom of polycarbonate bottles. This delivery rate turned over the volume of experimental bottles >100 times daily, ensuring that desired pCO_2_ concentrations and pH levels were generally maintained [3]. Bubbling was established two days before the beginning of each experiment to ensure that pCO_2_ concentrations and pH levels had reached a state of equilibrium and experiments persisted for ~ one week. Measurements of pH within bottles were made throughout each experiment using an Orion Star A321 Plus electrode (± 0.001) calibrated prior to each use using National Institute of Standards and Technology (NIST) traceable standards. Measurements using this pH meter were highly similar to and never significantly different from scale-adjusted spectrophotometric pH measurements made using *m*-cresol purple as described by Dickson et al. [30]. DIC concentrations in bottles were measured using an EGM-4 Environmental Gas Analyzer (PP Systems) system that quantifies DIC levels after separating the gas phase from seawater via acidification using a Liqui-Cel Membrane (Membrana) [3]. This instrument provided a methodological precision better than ± 1% for replicated measurements of total dissolved inorganic carbon. The levels of DIC and pH within Dr. Andrew Dickson’s (University of California, San Diego, Scripps Institution of Oceanography) certified reference material (Batch 138, 141) were measured during every analytical run as a quality assurance measure; analysis of samples proceeded only after complete recovery of certified reference material was attained. pCO_2_ levels (mean of t = initial and t = final, Table 1) were calculated using measured levels of DIC, pH (NIST), temperature, and salinity, as well as the first and second dissociation constants of carbonic acid in seawater according to Roy et al. [31] using the program CO2SYS (http://cdiac.ornl.gov/ftp/co2sys/). The targeted levels of pCO_2_ resulted in actual pCO_2_ and pH values of 441 ± 72 μatm and 7.9 ± 0.1, respectively, for ambient conditions and 1941 ± 141 μatm and 7.3 ± 0.1, respectively, for the elevated CO_2_ conditions (Table 1).

**Table 1:** Mean pH, temperature, salinity, pCO_2_, DIC, and alkalinity present during experiments and starting dissolved inorganic nitrogen (DIN), and dissolved inorganic phosphorus (DIP) concentrations during experiments.

*Gracilaria*										
Treatment	pH	Temperature	Salinity	pCO_2_	DIC	HCO_3_^-^	Alkalinity	DIN	DIP	
Control	8.23±0.02	18.4±0.1	29.5±0.8	327±58	1520±73	1380±73	1790±76	5.42±0.87	0.72±0.11	
Nutrients	8.29±0.03	18.5±0.1	29.5±0.7	314±61	1400±68	1310±93	1720±71	55.42±8.86	3.72±0.58	
CO_2_	7.37±0.01	18.6±0.1	29.8±0.6	2530±108	1760±60	1660±48	1710±59	5.42±0.87	0.72±0.11	
CO_2_/Nutrients	7.38±0.01	18.6±0.1	29.7±0.7	2380±114	1710±61	1630±49	1670±58	55.42±8.86	3.72±0.53	
*Ulva*										
Treatment	pH	Temperature	Salinity	pCO_2_	DIC	HCO_3_^-^	Alkalinity	DIN		DIP
Control	8.27±0.02	18.5±0.1	29.3±0.8	329±55	1540±72	1380±70	1780±76	5.42±0.87		0.72±0.11
Nutrients	8.35±0.03	18.5±0.1	29.6±0.7	328±56	1440±57	1330±63	1750±89	55.42±8.86		3.72±0.58
CO_2_	7.37±0.01	18.6±0.1	29.7±0.6	2510±102	1770±70	1650±56	1720±70	5.42±0.87		0.72±0.11
CO_2_/Nutrients	7.40±0.01	18.6±0.1	29.8±0.6	2300±163	1740±55	1650±46	1700±58	55.42±8.86		3.72±0.53

Mean values of pH (NBS scale), temperature (°C), salinity (g kg^-1^), pCO_2_ (μatm), DIC (μmol kgSW^-1^), HCO_3_^-^ (μmol kgSW^-1^), alkalinity (μmol kgSW^-1^), DIN (μM), and DIP (μM) for *Gracilaria* and *Ulva* for June through November experiments.

Values represent means ± SE. Data from individual experiments appear within S1 Table.

Experiments began with the addition of nutrients and introduction of macroalgae into experimental bottles. Experiments were maintained for seven days, during which daily pH and temperature measurements of each individual bottle were made with the Orion Star A321. Continuous measurements of light and temperature present during experiments were made using a LI-COR LI-1500 light sensor logger and HOBO pendant temperature and light data loggers and continuous pH measurements were made within selected bottles using the Orion Star A321 pH meter. At the termination of experiments, final pH and temperature measurements were made and a final DIC sample from each bottle was analyzed as described above. After measuring DIC, each macroalgae sample was removed from their respective bottles and rinsed, spun, re-rinsed, re-spun, and weighed as described above. *Gracilaria* samples were placed into small freezer bags for further analysis, whereas *Ulva* samples were placed between two transparency films and flattened with care to minimize folds. The surface areas of the samples were analyzed using SigmaScan Pro 5. Weight-based growth rates for both species were determined as described above. Significant differences in growth rates during experiments were assessed using a three-way ANOVA within SigmaPlot 11.0 where the main treatment effects were pCO_2_ treatment (ambient or elevated), nutrients (none or enhanced), and date of experiment.

### Tissue analyses

Identification of macroalgae was based on morphology, microscopy, known biogeography, and DNA sequencing. *Gracilaria tikvahiae* is one of the most common species of red algae along the North American east coast, is the only *Gracilaria* species native to the Northeast US [32–34], and displays a distinct, continuous, phylogenic lineage across the Canadian-Northeast-Mid-Atlantic US region [35]. The morphology and pigmentation of *Gracilaria* fronds used in this study were fully consistent with prior descriptions of *Gracilaria tikvahiae* in the region [32–33, 35] and this was considered to be the species of *Gracilaria* used during this study. In contrast to *Gracilaria*, identifying *Ulva* spp. across the Northeast US is more challenging due to the co-occurrence of multiple, morphologically similar species [36]. For this study, selected frozen *Ulva* samples were dried at 55°C and then homogenized into a fine powder using a mortar and pestle. DNA from selected samples were extracted using the CTAB method and the quality and quantity of nucleic acids were assessed by use of a Nanodrop 2000 spectrophotometer [29]. Next-generation DNA sequencing of ITS1 and ITS2 regions of the ribosome of samples [29, 36] was performed on extracted samples using an Illumina MiSeq at the Molecular Research Laboratory (Shallowater, TX, USA). Forward primer 18S1763 (5`-GGTGAACCTGCGGAGGGATCATT-3`) and reverse primer 5.8S142 (5`-TATTCCGACGCTGAGGCAG-3`) were used for amplification of ITS1 whereas for ITS2, forward primer 5.8S30 (5`-GCAACGATGAAGAACGCAGC-3`) and reverse primer ENT26S (5`-GCTTATTGATATGCTTAAGTTCAGCGGGT-3`) were used [29]. The sequences in samples (Genbank Accession #KU306346) had the greatest similarity with *Ulva rigida* which has been previously identified in NY estuaries [29] and the US Northeast [36] and are synonymous with other *Ulva* spp. (*Ulva lactuca var*. *rigida*). Due to the plastic nature of macroalgal taxonomic nomenclature as well as the high similarity in ITS sequences among *Ulva* species [36–37] for the purposes of this study, we refer to these algae simply as *Ulva* and for consistency, refer to *Gracilaria tikvahiae* as *Gracilaria*.

For carbon (C) and nitrogen (N) analyses, frozen samples were dried at 55°C, and then homogenized into a fine powder using a mortar and pestle. The total tissue nitrogen and carbon content of the homogenized samples were analyzed using a CE Instruments Flash EA 1112 elemental analyzer [38]. Samples were analyzed for δ^13^C signatures using an elemental analyzer interfaced to a Europa 20–20 isotope ratio mass spectrometer at the UC Davis Stable Isotope Facility [29]. Concentrations of nitrate, ammonium, and phosphate were measured using standard wet chemical methods [39].

Finally, isotope mixing models were developed to estimate the use of CO_2_ and HCO_3_^-^ during experiments. The models considered the δ^13^C and biomass of macroalgal tissue before and after experiments, the δ^13^C of the tanked gas used for experiments (-27.7‰), the δ^13^C of the marine CO_2_ and HCO_3_^-^ pool (-10‰ and 0‰, respectively; [40–42]), the fractionation of C during macroalgal uptake of CO_2_ and HCO_3_^-^ (-20‰ and -10‰, respectively; [40–42]), the fractionation of C during conversion of tanked CO_2_ bubbled into experimental vessels to HCO_3_^-^ (+10‰, respectively; [40–42]), and the concentration of DIC with and without the addition of tanked CO_2_ with the later providing an indication of the fraction of DIC contributed by the tanked gas compared to ambient air. We assumed that during the course of the experiment, the tanked CO_2_ gas came to equilibrium with the total DIC pool and thus that the HCO_3_^-^ pool took on a lighter δ^13^C signature in a manner proportional to the fraction of the DIC pool comprised of tanked gas compared to ambient air. Next, since we dried and homogenized the entire experimental macroalgal fronds for subsequent analyses, we assumed that the δ^13^C signature of the algal tissue was proportionally representative of the fraction of original tissue (pre-experiment) with its original δ^13^C signature and that the tissue grown during the experiment would take on a δ^13^C value representative of the CO_2_ or HCO_3_^-^ pool with a value made proportionally more negative by the tanked CO_2_. Finally, two sets of mixing models were run for each macroalgal species to estimate their δ^13^C signature based on using exclusively CO_2_ and exclusively HCO_3_^-^ during experiments. One-way ANOVAs were used to assess the differences between the measured δ^13^C signature of the macroalgae and signatures calculated based on exclusive CO_2_ or HCO_3_^-^ use and Tukey tests were used to assess differences between individual groups.

## Results

### Gracilaria

**Fig 2:**
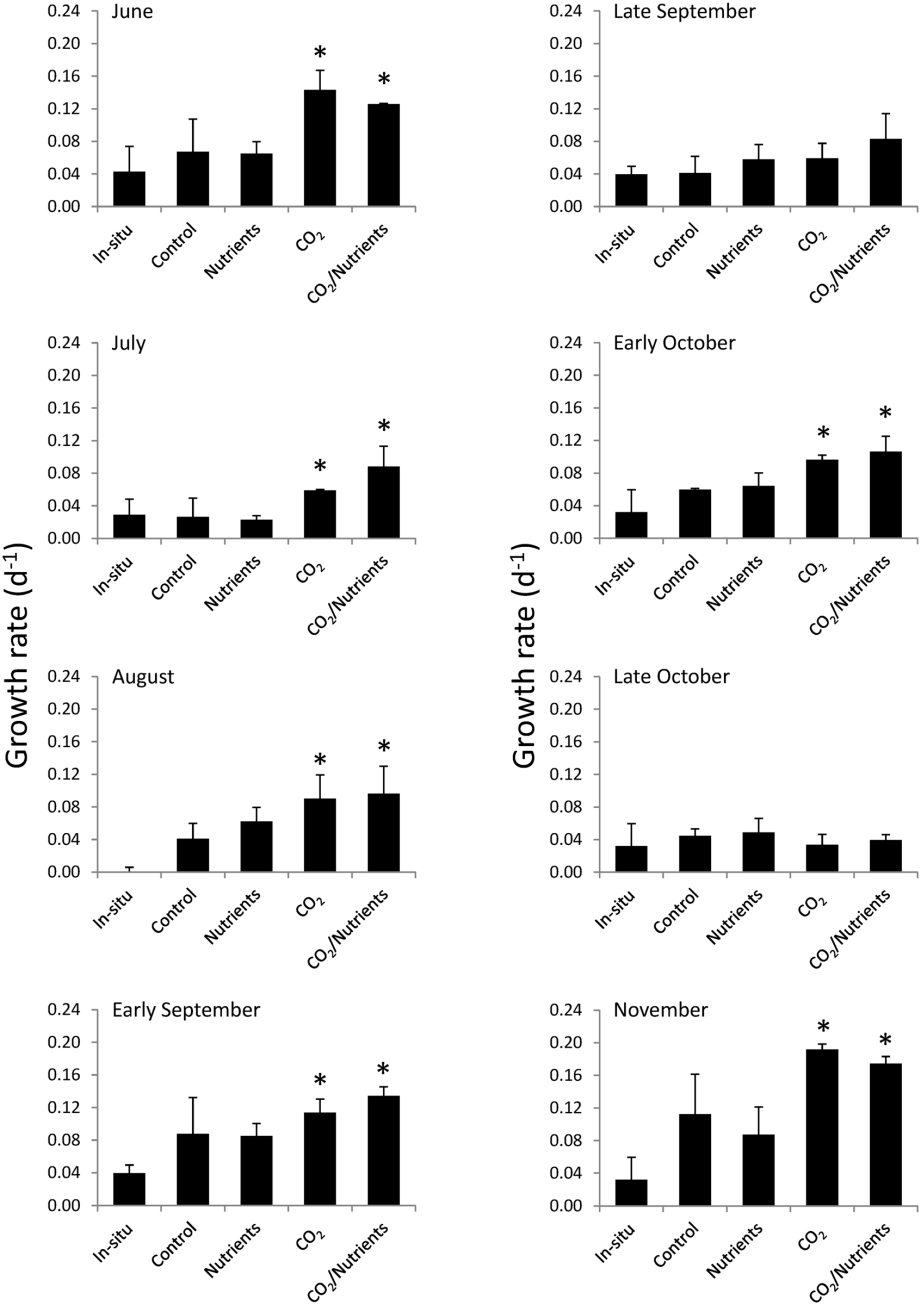
*Gracilaria* growth rates. Growth rates of *Gracilaria* exposed ambient and elevated CO_2_ conditions with and without nutrient additions for experiments performed August through November. Columns with an asterisk over them indicate significant results.

The in situ growth of *Gracilaria* in Shinnecock Bay was found to be highly similar to and not significantly different from growth rates within experimental control bottles with the exception of the August experiment, when experimental growth rates exceeded those in situ (Two-way ANOVA; *p >* 0.05; Fig 2; S2 Table). *Gracilaria* growth rates differed seasonally (Three-way ANOVA; *p* < 0.05; Fig 2; S2 Table). The experimental growth rates of *Gracilaria* were highly sensitive to changes in CO_2_ concentrations (Three-way ANOVA; *p* < 0.05; Fig 2; S2 Table). For six of the eight experiments, the growth of *Gracilaria* increased significantly when exposed to elevated CO_2_ concentrations (Tukey test; *p* < 0.05; Fig 2; S2 Table) with experiments during late September and late October being the exceptions to this trend. On average, growth rates under elevated CO_2_ were 70% higher than growth under ambient conditions (Fig 2). In contrast, the addition of nutrients did not significantly alter the growth rates of *Gracilaria* or yield statistically significant interactions with elevated pCO_2_ concentrations during any experiment (S2 Table).

**Fig 3:**
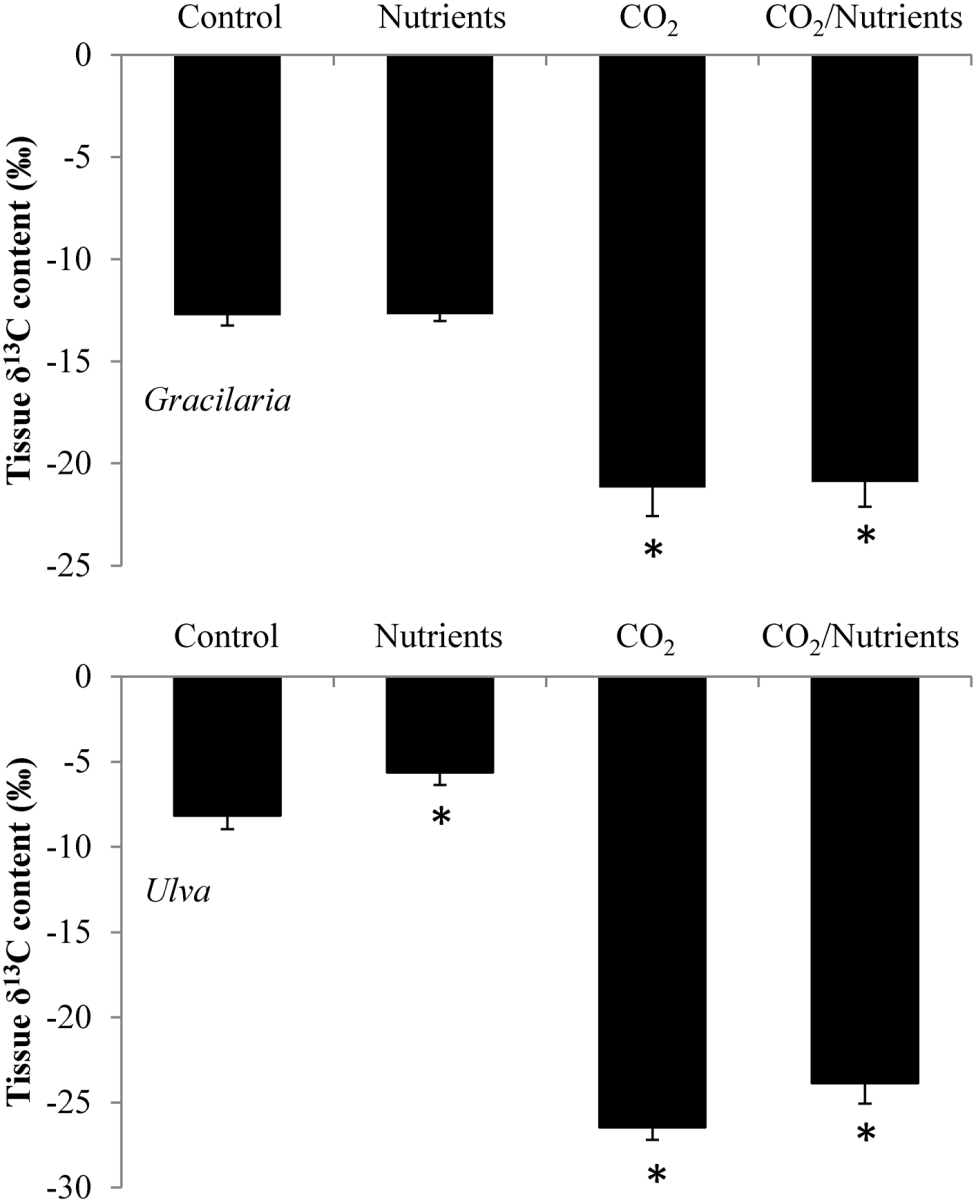
Macroalgal tissue δ^13^C. δ^13^C content of *Gracilaria* and *Ulva* exposed to ambient and elevated CO_2_ conditions with and without nutrient additions for experiments performed August through November.

**Fig 4:**
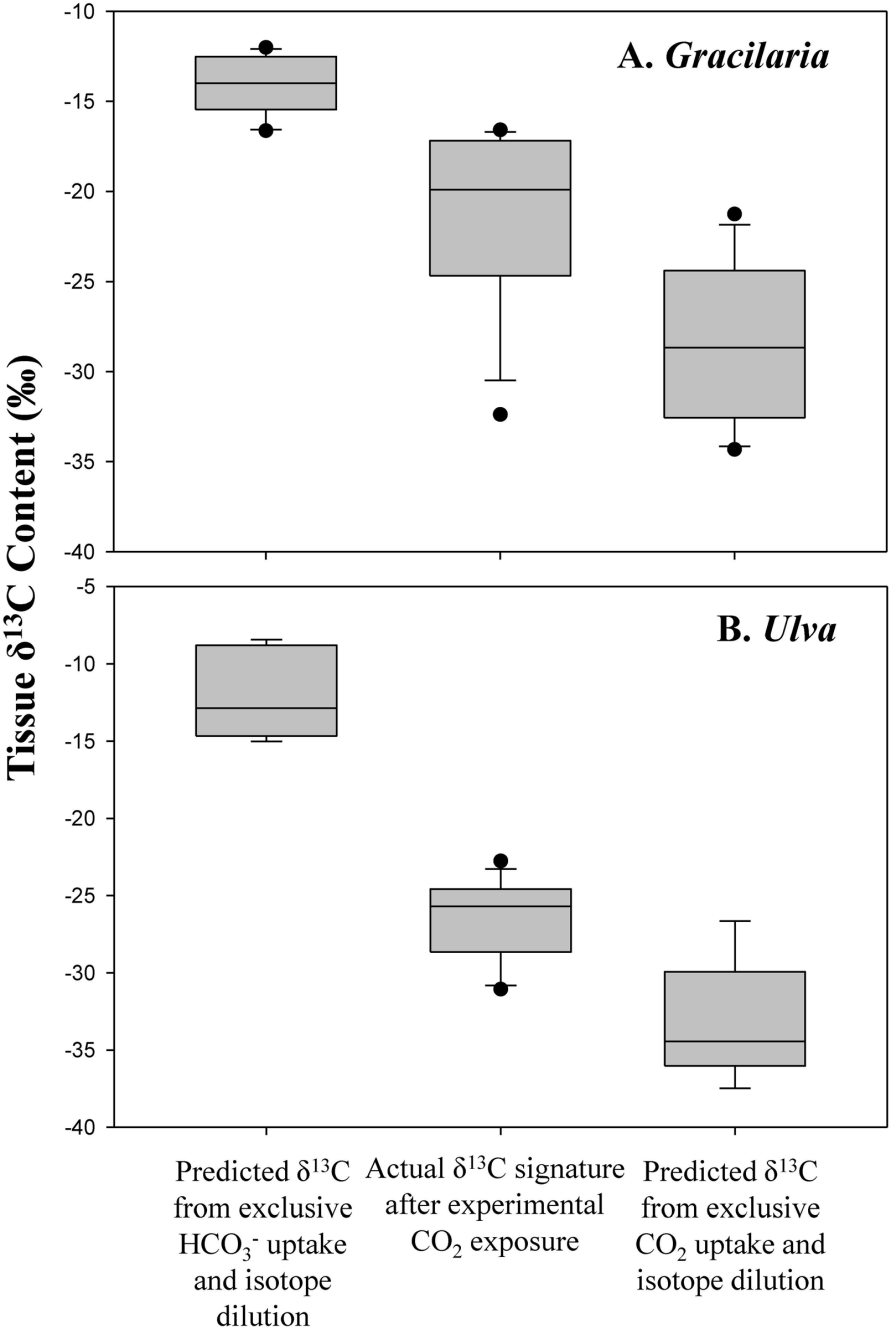
δ^13^C mixing model. δ^13^C content of A) *Gracilaria* and B) *Ulva* exposed to elevated CO_2_ conditions compared with the δ^13^C signature expected from the exclusive use of CO_2_ or the exclusive use of HCO_3_^-^. Box plots depict the mean median (line within the boxes), 25th and 75^th^ percentiles (lower and upper edges of the boxes), and 10th and 90^th^ percentiles of the data (lower and upper error bars).

The stable carbon isotope (δ^13^C) content of *Gracilaria* was significantly reduced by exposure to elevated pCO_2_, with the average δ^13^C value of the ambient and elevated CO_2_ groups being, on average, -13‰ and -21‰, respectively (Three-way ANOVA; *p*<0.001; Fig 3; S2 and S3 Tables). The δ^13^C signatures of *Gracilaria* were not altered by nutrients but did differ by experiment (Three-way ANOVA; *p*<0.001; S2 and S3 Tables). Isotope mixing models demonstrated that when incubated with elevated pCO_2_ concentrations, *Gracilaria* δ^13^C signatures (-21‰) were significantly lower than values expected if their DIC was exclusively from use of HCO_3_^-^ (-14‰) and significantly higher than expected from the use of exclusively CO_2_ (-28‰; Tukey test; *p*<0.001; Fig 4; S2 and S3 Tables). Quantitatively, the model suggested *Gracilaria* was using equal amounts of HCO_3_^-^ and CO_2_ during experimental incubations with CO_2_ (Fig 4).

**Fig 5:**
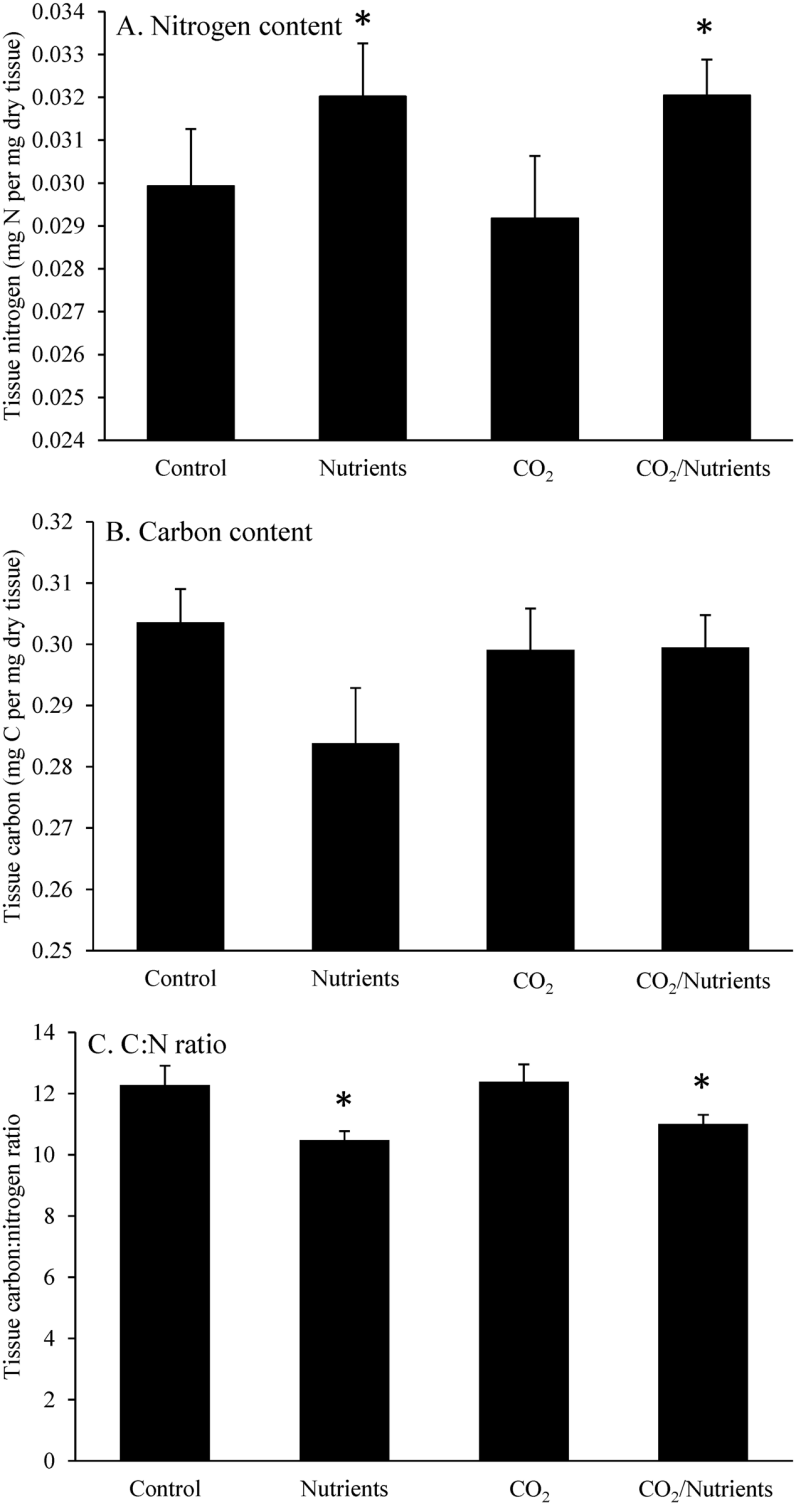
*Gracilaria* tissue nitrogen, carbon, and C:N. Tissue nitrogen, carbon, and C:N content of *Gracilaria* exposed to ambient and elevated CO_2_ conditions with and without nutrient additions for experiments performed August through November.

The nitrogen content of *Gracilaria* during experiments was found to be significantly higher in treatments that received nutrients and was found to differ seasonally (Three-way ANOVA; *p* < 0.05; S2 Table). On average, ambient and elevated nutrient treatments were found to have tissue nitrogen concentrations of 0.029 ± 0.005 and 0.032 ± 0.004 g N per g dry tissue, respectively (Fig 5; S4 Table). In contrast, the carbon content of *Gracilaria* was not significantly altered by pCO_2_ or nutrients, but did differ by seasonally (Three-way ANOVA; *p* < 0.05; Fig 5; S2 and S4 Tables). The tissue C:N ratio of *Gracilaria* was found to be significantly lower under elevated nutrient treatments (10.7 ± 0.2) compared to ambient nutrient treatments (12.3 ± 0.4) and differed seasonally (Three-way ANOVA; *p* < 0.05; Fig 5; S2 and S4 Tables). Tissue C:N ratio was not significantly changed in the CO_2_ treatments (S2 Table).

### Ulva

**Fig 6:**
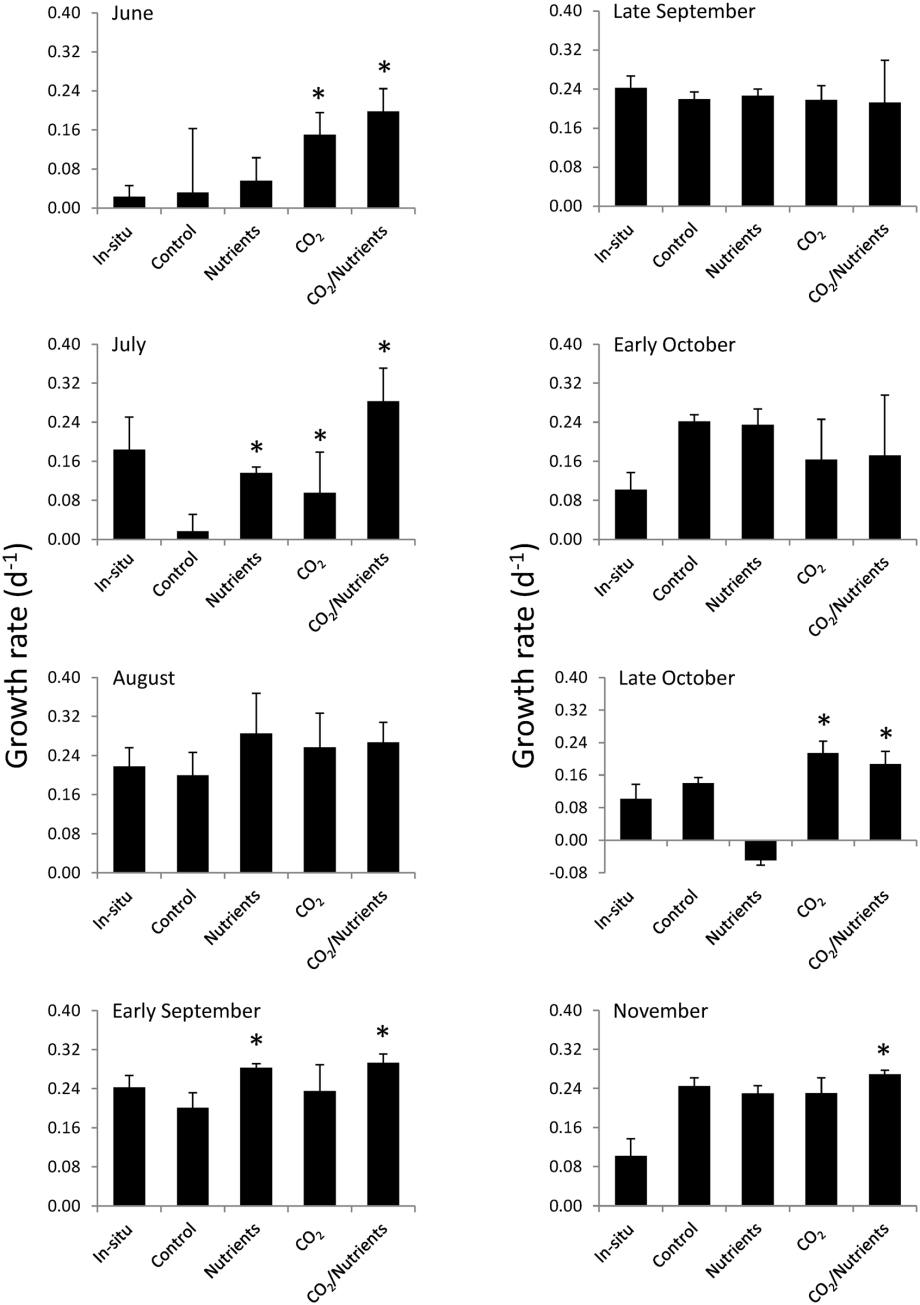
*Ulva* growth rates. Growth rates of *Ulva* exposed to ambient and elevated CO_2_ conditions with and without nutrient additions for experiments performed August through November. Columns with an asterisk over them indicate significant results.

The growth rates of *Ulva* during in situ experiments did not differ statistically from those found within experimental control bottles except during experiments in early October and November when experimental control growth rates were greater than those observed in situ (Two-way ANOVA; *p* > 0.05; Fig 6; S2 Table). *Ulva* growth rates differed by experiment (Three-way ANOVA; *p* < 0.05; Fig 5; S2 Table). *Ulva* displayed more complex responses to nutrients and CO_2_ concentrations during experiments compared to *Gracilaria*. During experiments in June, July, and late October, *Ulva* growth rates significantly increased in response to elevated CO_2_ concentrations (Tukey test; *p* < 0.05; Fig 6; S2 Table). In addition, *Ulva* experienced significantly higher growth rates in response to higher nutrient levels during experiments performed during July and early September (Fig 6; Tukey test; *p* < 0.05; S2 Table). Finally, there was an interactive effect of CO_2_ and nutrients during the late October and November experiments during which these two factors synergistically increased the growth rates of *Ulva* (*p* < 0.05; S2 Table). On average, for all experiments, *Ulva* growth rates when exposed to elevated CO_2_ were 30% higher than ambient conditions (Fig 5; *p*<0.05; Three-way ANOVA; S2 Table) whereas the nutrients yielded a smaller, non-significant increase in growth rates (13%; Fig 6).

In a manner similar to *Gracilaria*, the δ^13^C content of *Ulva* was significantly reduced by exposure to elevated pCO_2_ (to -27‰) relative to control treatments value of -7‰ (Three-way ANOVA; *p*<0.001; Fig 3; S2 and S3 Tables). Unlike *Gracilaria*, however, the δ^13^C of *Ulva* was also effected by nutrients that yielded significantly higher values (-5‰) relative to control treatments (-7‰) and the δ^13^C differed by experiment (Three-way ANOVA; *p*<0.05; Fig 3; S2 and S3 Tables). Nutrients and CO_2_ did not interact to alter the δ^13^C of *Ulva*. Isotope mixing models indicated that when incubated with elevated pCO_2_ concentrations, *Ulva* δ^13^C signatures (-27‰) were significantly lower than values expected from the exclusively use of HCO_3_^-^ (-12‰) and significantly higher than expected from the use of exclusively CO_2_ (-33‰; Tukey test; *p*<0.001; Fig 4; S2 Table). Quantitatively, the model suggested that for *Ulva*, during experimental incubations with elevated CO_2_, ~70% of their carbon came from CO_2_ and ~30% came from HCO_3_^-^ (Fig 4).

**Fig 7:**
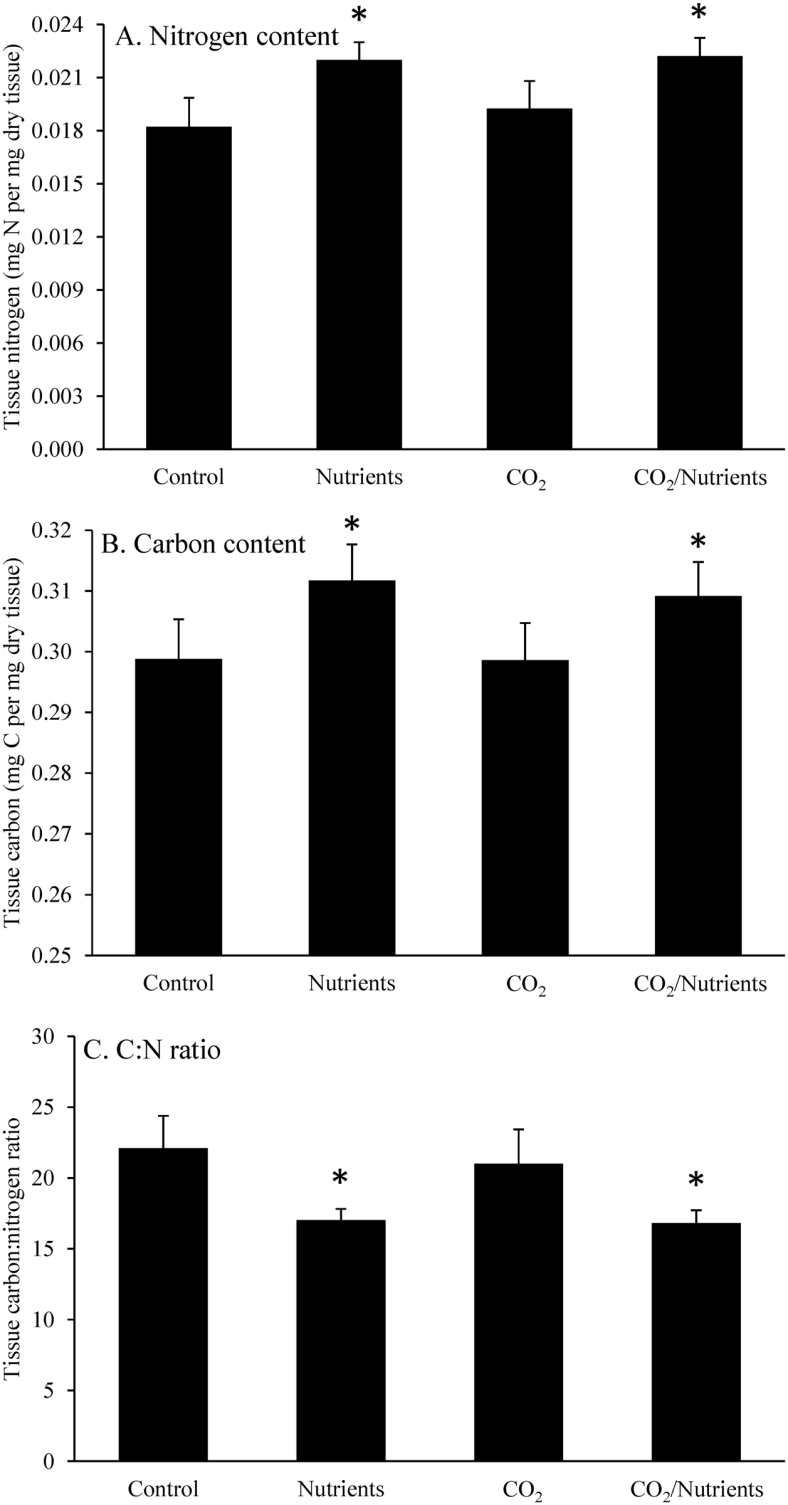
*Ulva* tissue nitrogen, carbon, and C:N. Tissue nitrogen, carbon, and C:N content of *Ulva* exposed to ambient and elevated CO_2_ conditions with and without nutrient additions for experiments performed August through November.

Also similar to *Gracilaria*, the nitrogen content of *Ulva* was significantly higher in elevated nutrient treatments (0.022 ± 0.004 g N per g dry tissue) compared to ambient nutrient treatments, regardless of pCO_2_ concentrations (0.019 ± 0.006 g N per g dry tissue; Three-way ANOVA; *p* < 0.05; Fig 7; S2 and S4 Tables). The carbon content of *Ulva* was not significantly altered by CO_2_ but was significantly increased by nutrients and differed by experiment (Three-way ANOVA; *p* < 0.05; Fig 7; S2 and S4 Tables). Tissue C:N was significantly lower in the elevated nutrient treatments (16.9 ± 0.6) than ambient nutrient treatments (21.5 ± 1.6) and differed by experiment (Three-way ANOVA; *p* < 0.05; Fig 7; S2 and S4 Tables).

## Discussion

During this study, elevated levels of pCO_2_ were found to significantly enhance the growth rates of two bloom-forming, estuarine macroalgae, *Gracilaria* and *Ulva*. These enhanced growth rates were accompanied by large and significant reductions in the δ^13^C content of the macroalgae. Concurrently, nutrients were found to enhance the growth of *Ulva* but not *Gracilaria*, and the combination of elevated nutrients and pCO_2_ were capable of synergistically promoting the growth of *Ulva*. Given that elevated pCO_2_ and acidification of coastal ecosystems are symptoms of eutrophication and that ocean acidification is enriching pCO_2_ concentrations in these systems, this study provides new insight regarding the present and future overgrowth of macroalgae in estuaries.

The effects of elevated CO_2_ concentrations on the growth of algae can depend on the precise carbon acquisition pathways utilized. C_3_ algae can benefit from high CO_2_ as their RuBisCO is not substrate-saturated at current CO_2_ levels (~400ppm) [7, 43]. Many macroalgae use HCO_3_^-^ rather than dissolved CO_2_ under current seawater pCO_2_ concentrations and utilize CA to convert HCO_3_^-^ to CO_2_ for use by RuBisCO [1, 8–10]. For example, Mercado et al. [44] found that the chlorophytes *Ulva rigida* and *U*. *compressa* (formerly *Enteromorpha*) do not receive enough CO_2_ through diffusive uptake alone at current CO_2_ levels and thus must use CCMs to acquire HCO_3_^-^. However, when exposed to elevated pCO_2_, macroalgae may down-regulate their CCMs, reduce the use of HCO_3_^-^, and begin to rely on CO_2_ as a primary C source [19, 45–47]. The energy made available from the down-regulation of the CCM may, in turn, be used for other purposes, such as increased vegetative growth [1] which we observed during this study.

Values of δ^13^C are often used to assess the types of carbon utilized by macroalgae. The δ^13^C of HCO_3_^-^ is significantly higher (less negative) than that of CO_2_ in seawater and values of -10‰ or higher in macroalgae are reflective of the sole use HCO_3_^-^ and CCMs whereas macroalgae relying wholly on diffusion of CO_2_ for carbon attain a value of -30‰ [40–41, 48]. When grown in ambient seawater, *Ulva* and *Gracilaria* had δ^13^C values of -8 and -13‰, values indicative of exclusive and near exclusive (85%) HCO_3_^-^ use, respectively [40–41, 48]. The use of tanked CO_2_ gas with a known δ^13^C signature (-27.7‰) permitted that CO_2_ to be used as a tracer in mixing models and demonstrated that when incubated with elevated CO_2_, both macroalgal species switched their primary source of DIC. For *Ulva*, the change was the most dramatic as the three-fold decrease in δ^13^C signature was indicative of these algae going from exclusive use of HCO_3_^-^ to, on average, 70% of their DIC originating from CO_2_ and only 30% from HCO_3_^-^. For *Gracilaria*, the change was less dramatic with but still notable as the alga went from ~85% HCO_3_^-^ use under low pCO_2_ conditions to 50% CO_2_ use under high pCO_2_ conditions. Given the switch to increasing CO_2_ use by *Ulva* and *Gracilaria* and concurrent increase in growth experienced under elevated pCO_2_ concentrations, these algae may have down-regulated their CCMs permitting more energy to be dedicated to vegetative growth [1]. The significant increase in δ^13^C of *Ulva* when provided with nutrients further supports these hypotheses given that they experienced enhanced growth and presumably greater photosynthetic rates due to higher nutrient levels, causing a greater use of HCO_3_^-^ via CCMs since additional CO_2_ was not available [21]. Finally, there are additional factors that could contribute to lowered δ^13^C values including preferential synthesis of lipids depleted in δ^13^C compared to proteins and carbohydrates [49] although the extent of fractionated associated with this process is small compared to changes observed during experiments presented here. Hence, the change in δ^13^C values during experiments suggest that when exposed to high concentrations of CO_2_, these bloom-forming macroalgae obtained a significantly larger fraction of their DIC from CO_2_ and often grew faster.

Elevated pCO_2_ concentrations did not alter the rate at which macroalgae took up and stored carbon (C) or nitrogen (N). The lack of change in tissue C content is consistent with the findings of Gordillo et al. [22] who reported no accumulation of soluble carbohydrates and no change in tissue C content for *Ulva rigida* fronds exposed to pCO_2_-enriched conditions. Despite the unchanged tissue C content, there were expected, significant increases in tissue N content within nutrient treatments. Both *Ulva* and *Gracilaria* have been shown to be able to rapidly assimilate and store nitrate [50–51] and have been shown to experience enhanced tissue N content when exposed to elevated levels of nitrate [28, 52]. While increases in the C:N ratio of macroalgae can reflect an increase in soluble carbohydrates during stimulation of growth rates in certain plants [53], during our study tissue C:N levels did not track growth rates. Given the observed changes in δ^13^C during exposure to high pCO_2_, we hypothesize that macroalgae responded to increased C availability by increasing, stoichiometrically-balanced growth rather than by storing more carbohydrates.

Eutrophication has been shown to promote coastal ocean acidification due to the accumulation of respiratory CO_2_ emanating from the microbial degradation of the excessive organic matter [16]. The present study has shown that *Gracilaria* and *Ulva* are capable of enhanced growth under elevated pCO_2_ levels and that *Ulva* can, on occasion, synergistically benefit from concurrently higher nutrient concentrations. Going forward, this finding may have broad implications as it demonstrates that, in some cases, the true impacts of elevated pCO_2_ on macroalgae may only be realized when excessive nutrients are present. Prior studies have demonstrated that elevated CO_2_ levels may have little effect on photosynthetic rates of some algae [10, 19, 47] but can result in increased biomass of *Gracilaria* sp., *G*. *chilensis*, and *G*. *lemaneiformis* [45–46] and *Ulva rigida* and *U*. *lactuca* [20, 22]. While *Gracilaria* can benefit from high nutrient concentrations [26], *Ulva* is capable of undergoing more rapid growth in eutrophic settings [29] due to a high maximum rate of uptake of ammonium and nitrate [17]. This was observed during the present study as *Ulva* growth rates were significantly higher than *Gracilaria*, and *Ulva* responded to nutrients more consistently than *Gracilaria*. *Ulva* is known to outcompete slower-growing algae in eutrophic estuaries, such as Saldanha Bay, South Africa [54], Britanny, France [25], and Qingdao, China [23]. The current study demonstrates that within eutrophied estuaries, seasonally elevated levels of pCO_2_ may be equally or more important than excessive nutrients in promoting algal growth. For example, *Gracilaria* grew faster in the presence of higher pCO_2_ levels but was unaffected by nutrients. Previously, it has been noted that more pristine estuaries are characterized by numerous, slower-growing macroalgal species while eutrophic estuaries are typically dominated by fewer, fast-growing, ephemeral macroalgal species [17–18, 24]. While nutrient loading and changes in light levels have been ascribed as the factors controlling these trends, the findings presented here suggest that elevated levels of pCO_2_ may be equally or more important for shaping estuarine macroalgal community composition.

The extent to which elevated levels of pCO_2_ affect the growth of macroalgae in estuaries will likely be influenced, in part, by physical mixing and circulation. In poorly flushed and/or mixed estuarine regions, diffusive boundary layers around seaweeds may limit DIC uptake [55–56] and thus higher ambient pCO_2_ may be more likely to be beneficial. In contrast, in high energy environments with fast-moving currents or wave-flow, boundary layers are less likely to develop [55–56] and additional pCO_2_ may be less likely to affect growth. During this study, macroalgae were vigorously bubbled at a rate that turned over the dissolved gas pool more than 700-times daily, a process that was unlikely to permit the development of boundary layers. This hypothesis is supported by the highly similar growth rates of thalli in a fairly high energy region of Shinnecock Bay during in situ experiments and in our control, experimental bottles for nearly all experiments. Hence, in our experiments, enhanced growth experienced during exposure to high levels of pCO_2_ were more likely a consequence of an intra-cellular, photosynthetic benefit for the algae rather than changes in external conditions.

The full implications of climate change for macroalgal communities are not fully understood, as studies of the effects of processes such as ocean acidification, rising temperatures, and changes in nutrient loading rates have been performed on a limited number of species. Porzio et al. [57] examined >100 species of macroalgae near volcanic CO_2_ vents in the Gulf of Naples, Italy, and found 20 species of calcium carbonate-containing macroalgae were no longer present under the acidification, whereas the ochryophyte *Dictyota dichotoma* and the rhodophyte *Hildenbrandia rubra* were most abundant within the high CO_2_ environment. Other studies have similarly found that tropical calcifying macroalgae indigenous to coral reefs are likely to be negatively impacted by ocean acidification [58–60]. Connell and Russell [61] found that elevated CO_2_ and temperature enhanced the growth of opportunistic turf-forming algae and that expansion of this algae inhibited the growth of kelp (*Ecklonia radiata*). As climate change processes promote increased pCO_2_, this and prior studies suggest that macroalgal communities may shift and favor rapid-growing and opportunistic species such as *Ulva*, *Gracilaria*, and turf algae, perhaps to the detriment of calcifying macroalgae and/or kelp.

The more rapid growth of some species of macroalgae will have important implications for other classes of marine autotrophs. The majority of seagrass species are C_3_ plants that are not currently substrate-saturated at current CO_2_ levels, with some, such as *Zostera marina*, showing enhanced photosynthesis and growth under elevated CO_2_ concentrations [1, 5]. Elevated nutrient loading, however, typically favors the dominance of macroalgae over seagrasses, as macroalgae are more competitive for high nutrient levels and can overgrow and shade seagrass [18]. Beyond CO_2_, climate change-induced warming may further favor macroalgae among submerged aquatic vegetation as many temperate species of seagrass exist at or near their upper level of thermal tolerance [62]. Finally, although highly excessive nutrient loading in estuaries with extended residence times are thought to ultimately favor the growth of phytoplankton blooms over macroalgae, the ability of both *Ulva* and *Gracilaria* to allelopathically inhibit the growth of phytoplankton [63–64] may allow macroalgae to remain dominant in high nutrient, high CO_2_ estuaries.

Macroalgal blooms can be harmful to marine life. Specifically, the overgrowth of macroalgae can cover critical benthic habitats and promote diel hypoxia/anoxia in estuaries [18, 28, 65] and *Ulva* has been shown to cause mortality in multiple calcifying animals including bivalves, barnacles, and larval crabs [27, 66–67]. Since these calcifying animals are also sensitive to high levels of CO_2_ [3, 68–70] the stimulation of harmful macroalgae such as *Ulva* under elevated pCO_2_ levels may represent a previously unrecognized, compounding environmental threat to some ocean animals. In contrast, other animals might benefit from predicted shifts in macroalgal communities. Some herbivorous fish of the families Blenniidae, Kyphosidae, and Siganidae selectively feed on filamentous and fleshy seaweeds such as *Ulva* [71] and *Ulva lactuca* can be an important nursery for juvenile blue crabs (*Cahnectes sapidus*) [72]. Furthermore, excessive nutrient loading generally enhances the nitrogen content and C:N ratio of macroalgal tissues, which could benefit herbivores feeding on such material [73]. Hence, while shifts in macroalgal communities caused by climate change and eutrophication may promote the prevalence of non-calcifying macroalgae over seagrasses and calcifying macroalgae and may be harmful to some marine mollusks, these shifts could benefit marine organisms that either graze on macroalgae or utilize it as a nursery.

## Conclusion

This study demonstrated that two species of bloom-forming macroalgae experience fundamental changes in their photosynthetic physiology when exposed to high, but coastally realistic levels of pCO_2_ that led to significantly enhanced growth rates. Regarding *Ulva*, concurrently enhanced pCO_2_ and nutrient levels yielded synergistically increased growth. More studies are needed to understand the extent to which this phenomenon is applicable to other estuarine chlorophytes and rhodophytes as well as the ecosystem-wide implications of this phenomenon. Regardless, given that eutrophication can yield elevated levels of pCO_2_, this study suggests that the overgrowth of macroalgae in eutrophic estuaries may be promoted by acidification, a process that will intensify in coming decades.

## Supporting Information

S1 Table Values of pH (NBS scale), temperature (°C), salinity (g kg^-1^), and pCO_2_ (μatm) for *Gracilaria* and *Ulva* for June through November experiments. Values represent means ± SE.
(PDF)

S2 Table Statistical analyses of variance for laboratory and in situ experiments (June through November 2014) for *Gracilaria* and *Ulva*. (PDF)

S3 Table Tissue δ^13^C content (‰) of dry tissue samples of *Gracilaria* and *Ulva* for August through November experiments. Values represent means ± SE.
(PDF)

S4 Table Tissue nitrogen content (g N per g dry tissue), tissue carbon content (g N per g dry tissue), and tissue C:N of dry tissue samples of *Gracilaria* and *Ulva* for August through November experiments. Values represent means ± SE.
(PDF)
