# Supplementary material for: Ocean Acidification Accelerates the Growth of Two Bloom-Forming Macroalgae
Source: PLoS One. 2016 May 13;11(5):e0155152. doi: 10.1371/journal.pone.0155152 (PMC4866684; doi:10.1371/journal.pone.0155152)
Supplement: S1 Table — Values represent means ± SE. (PDF) [file pone.0155152.s001.pdf]

Supporting Information for: Ocean acidification accelerates the growth of two bloom-forming, estuarine macroalgae

Craig S. Young and Christopher J. Gobler

Supplementary Tables

S1 Tables. Values of pH (NBS scale), temperature (°C), salinity (g kg^{-1}), and pCO_2 (µatm) for *Gracilaria* and *Ulva* for June through November experiments. Values represent means ± SE.

Gracilaria

June

Treatment	pH	Temperature	Salinity
Control	8.31±0.09	18.7±0.1	31.1±0.1
Nutrients	8.38±0.09	18.7±0.1	31.1±0.1
CO_2	7.44±0.01	18.7±0.1	31.1±0.1
CO_2/Nutrients	7.44±0.01	18.7±0.1	31.1±0.1

July

Treatment	pH	Temperature	Salinity	pCO_2
Control	8.28±0.04	18.8±0.1	30.8±0.8	377±149
Nutrients	8.48±0.09	18.7±0.1	30.6±0.6	287±240
CO_2	7.40±0.02	18.7±0.1	30.8±0.8	3120±97
CO_2/Nutrients	7.42±0.03	18.7±0.1	30.8±0.8	3210±0.6

August

Treatment	pH	Temperature	Salinity	pCO_2
Control	8.20±0.04	18.4±0.1	31.0±0.1	296±212
Nutrients	8.31±0.07	18.5±0.1	31.0±0.1	343±163
CO_2	7.39±0.03	18.6±0.1	31.0±0.1	2340±92
CO_2/Nutrients	7.40±0.02	18.7±0.1	31.0±0.1	2030±457

Early September

Treatment	pH	Temperature	Salinity	pCO_2
Control	8.20±0.05	18.6±0.1	31.4±0.1	310±201
Nutrients	8.24±0.06	18.6±0.1	31.5±0.1	306±212
CO_2	7.37±0.01	18.7±0.1	31.4±0.1	2570±26
CO_2/Nutrients	7.41±0.02	18.6±0.1	31.3±0.1	1640±220

Late September

Treatment	pH	Temperature	Salinity	pCO_2
Control	8.30±0.08	18.6±0.1	32.1±0.1	317±275
Nutrients	8.26±0.07	18.6±0.1	32.1±0.1	316±276
CO_2	7.33±0.01	18.8±0.1	32.1±0.1	2220±70
CO_2/Nutrients	7.34±0.02	18.7±0.1	32.1±0.1	2210±64

Early October

Treatment	pH	Temperature	Salinity	pCO_2
Control	8.20±0.06	18.3±0.6	25.4±3.4	324±224
Nutrients	8.21±0.06	19.0±0.1	25.7±3.7	303±223
CO_2	7.33±0.01	19.1±0.1	27.2±2.4	2770±391
CO_2/Nutrients	7.34±0.01	19.1±0.1	26.5±3.5	2610±674

Late October

Treatment	pH	Temperature	Salinity	pCO_2
Control	8.11±0.05	17.8±0.2	28.7±0.2	350±215
Nutrients	8.12±0.05	17.8±0.2	28.9±0.8	478±219
CO_2	7.31±0.01	17.9±0.2	28.5±0.1	2330±78
CO_2/Nutrients	7.32±0.01	17.9±0.2	29.1±0.5	2380±116

November

Treatment	pH	Temperature	Salinity	pCO_2
Control	8.22±0.06	17.8±0.1	26.1±0.3	334±147
Nutrients	8.28±0.07	17.7±0.1	26.6±0.1	307±151
CO_2	7.37±0.02	18.0±0.1	27.4±0.1	2460±205
CO_2/Nutrients	7.35±0.01	18.0±0.1	27.3±0.2	2340±107

Ulva

June

Treatment	pH	Temperature	Salinity
Control	8.37±0.08	18.7±0.1	31.1±0.1
Nutrients	8.42±0.09	18.7±0.1	31.1±0.1
CO_2	7.48±0.02	18.7±0.1	31.1±0.1
CO_2/Nutrients	7.46±0.01	18.7±0.1	31.1±0.1

July

Treatment	pH	Temperature	Salinity	pCO_2
Control	8.23±0.05	18.7±0.1	30.8±0.8	385±142
Nutrients	8.59±0.12	18.8±0.1	30.8±0.8	271±256
CO_2	7.40±0.02	18.7±0.1	30.7±0.7	3080±132
CO_2/Nutrients	7.45±0.03	18.7±0.1	30.8±0.8	2910±300

August

Treatment	pH	Temperature	Salinity	pCO_2
Control	8.27±0.03	18.5±0.1	31.0±0.1	283±141
Nutrients	8.35±0.06	18.4±0.1	31.0±0.1	321±119
CO_2	7.39±0.02	18.6±0.1	31.0±0.1	2110±257
CO_2/Nutrients	7.41±0.02	18.8±0.1	31.0±0.1	2130±446

Early September

Treatment	pH	Temperature	Salinity	pCO_2
Control	8.29±0.05	18.6±0.1	31.4±0.2	310±187
Nutrients	8.44±0.12	18.6±0.1	31.5±0.1	305±227
CO_2	7.38±0.02	18.7±0.1	31.4±0.1	2570±291
CO_2/Nutrients	7.50±0.05	18.6±0.1	31.3±0.3	1635±981

Late September

Treatment	pH	Temperature	Salinity	pCO_2
Control	8.37±0.08	18.6±0.1	32.1±0.1	317±275
Nutrients	8.40±0.09	18.6±0.1	32.1±0.1	316±276
CO_2	7.33±0.02	18.7±0.1	32.1±0.1	2220±69
CO_2/Nutrients	7.38±0.03	18.7±0.1	32.1±0.1	2210±64

Early October

Treatment	pH	Temperature	Salinity	pCO_2
Control	8.25±0.06	19.0±0.1	25.4±3.4	324±227
Nutrients	8.22±0.05	18.9±0.1	25.7±3.7	303±181
CO_2	7.32±0.01	19.1±0.1	27.2±1.8	2770±306
CO_2/Nutrients	7.32±0.01	19.0±0.1	26.5±2.9	2610±401

Late October

Treatment	pH	Temperature	Salinity	pCO_2
Control	8.17±0.04	17.8±0.2	28.7±0.7	350±207
Nutrients	8.09±0.03	17.9±0.2	28.9±0.2	478±59
CO_2	7.30±0.01	17.9±0.2	28.5±0.5	2330±70
CO_2/Nutrients	7.29±0.01	17.9±0.2	29.1±0.7	2380±29

November

Treatment	pH	Temperature	Salinity	pCO_2
Control	8.21±0.04	17.7±0.1	26.1±0.3	334±151
Nutrients	8.27±0.06	17.8±0.1	26.6±0.2	307±178
CO_2	7.34±0.02	17.8±0.1	27.4±0.1	2460±2
CO_2/Nutrients	7.38±0.02	18.0±0.1	27.3±0.1	2340±122
