# Supplementary material for: Ocean Acidification Accelerates the Growth of Two Bloom-Forming Macroalgae
Source: PLoS One. 2016 May 13;11(5):e0155152. doi: 10.1371/journal.pone.0155152 (PMC4866684; doi:10.1371/journal.pone.0155152)
Supplement: S2 Table — (PDF) [file pone.0155152.s002.pdf]

Supporting Information for: Ocean acidification accelerates the growth of two bloom-forming, estuarine macroalgae

Craig S. Young and Christopher J. Gobler

Supplementary Tables

S2 Tables. Statistical analyses of variance for laboratory and in situ experiments (June through November 2014) for *Gracilaria* and *Ulva*.

Three-way analysis of variance for *Gracilaria* growth for June through November experiments

Source of Variation	DF	SS	MS	F	P
CO_2	1	0.0385	0.0385	77.274	<0.001
Nutrients	1	0.000486	0.000486	0.974	0.328
Time	7	0.0921	0.0132	26.367	<0.001
CO_2 x Nutrients	1	0.000204	0.000204	0.409	0.525
CO_2 x Time	7	0.0168	0.0024	4.807	<0.001
Nutrients x Time	7	0.00388	0.000554	1.111	0.368
CO_2 x Nutrients x Time	7	0.00142	0.000202	0.406	0.895
Residual	60	0.0299	0.000499		
Total	91	0.184	0.00203		

Three-way analysis of variance for *Ulva* growth for June through November experiments

Source of Variation	DF	SS	MS	F	P
CO_2	1	0.0469	0.0469	17.413	<0.001
Nutrients	1	0.00887	0.00887	3.295	0.075
Time	7	0.239	0.0342	12.704	<0.001
CO_2 x Nutrients	1	0.00386	0.00386	1.432	0.236
CO_2 x Time	7	0.109	0.0156	5.805	<0.001
Nutrients x Time	7	0.077	0.011	4.085	0.001
CO_2 x Nutrients x Time	7	0.0228	0.00326	1.21	0.312
Residual	57	0.153	0.00269		
Total	88	0.659	0.00748		

Two-way analysis of variance for *Gracilaria* growth under control and in situ conditions

Source of Variation	DF	SS	MS	F	P
Location	1	0.00158	0.00158	2.844	0.104
Time	7	0.00507	0.00072	1.303	0.288
Location x Time	7	0.0208	0.00297	5.347	<0.001
Residual	26	0.0144	0.00056		
Total	41	0.0427	0.00104		

Two-way analysis of variance for *Ulva* growth under control and in situ conditions

Source of Variation	DF	SS	MS	F	P
Location	1	0.00068	0.00068	0.35	0.559
Time	7	0.203	0.029	14.847	<0.001
Location x Time	7	0.252	0.0359	18.398	<0.001
Residual	31	0.0606	0.00195		
Total	46	0.519	0.0113		

Three-way analysis of variance of the tissue nitrogen for *Gracilaria* for June through November experiments

Source of Variation	DF	SS	MS	F	P
CO_2	1	2.4E-06	2.4E-06	0.199	0.658
Nutrients	1	0.00011	0.00011	9.328	0.004
Time	5	0.00113	0.00023	18.989	<0.001
CO_2 x Nutrients	1	2.7E-06	2.7E-06	0.229	0.634
CO_2 x Time	5	7.5E-05	1.5E-05	1.26	0.297
Nutrients x Time	5	5.8E-05	1.2E-05	0.97	0.446
CO_2 x Nutrients x Time	5	4.1E-05	8.2E-06	0.694	0.63
Residual	48	0.00057	1.2E-05		
Total	71	0.00198	2.8E-05		

Three-way analysis of variance of the tissue carbon for *Gracilaria* for June through November experiments

Source of Variation	DF	SS	MS	F	P
CO_2	1	0.00056	0.00056	1.103	0.299
Nutrients	1	0.00168	0.00168	3.334	0.074
Time	5	0.0182	0.00363	7.183	<0.001
CO_2 x Nutrients	1	0.00183	0.00183	3.621	0.063
CO_2 x Time	5	0.00296	0.00059	1.171	0.337
Nutrients x Time	5	0.00247	0.0005	0.978	0.441
CO_2 x Nutrients x Time	5	0.00836	0.00167	3.308	0.012
Residual	48	0.0243	0.00051		
Total	71	0.0603	0.00085		

Three-way analysis of variance of the tissue C:N for *Gracilaria* for June through November experiments

Source of Variation	DF	SS	MS	F	P
CO_2	1	1.841	1.841	1.618	0.209
Nutrients	1	45.616	45.616	40.098	<0.001
Time	5	177.656	35.531	31.233	<0.001
CO_2 x Nutrients	1	0.796	0.796	0.7	0.407
CO_2 x Time	5	3.425	0.685	0.602	0.698
Nutrients x Time	5	32.098	6.42	5.643	<0.001
CO_2 x Nutrients x Time	5	2.763	0.553	0.486	0.785
Residual	48	54.606	1.138		
Total	71	318.799	4.49		

Three-way analysis of variance of the tissue nitrogen for *Ulva* for June through November experiments

Source of Variation	DF	SS	MS	F	P
CO_2	1	1.9E-06	1.9E-06	0.22	0.642
Nutrients	1	0.00018	0.00018	20.333	<0.001
Time	5	0.00097	0.0002	22.413	<0.001
CO_2 x Nutrients	1	4.7E-07	4.7E-07	0.054	0.817
CO_2 x Time	5	1.8E-05	3.6E-06	0.414	0.837
Nutrients x Time	5	0.00053	0.00011	12.147	<0.001
CO_2 x Nutrients x Time	5	1.4E-05	2.8E-06	0.317	0.9
Residual	44	0.00038	8.7E-06		
Total	67	0.00214	3.2E-05		

Three-way analysis of variance of the tissue carbon for *Ulva* for June through November experiments

Source of Variation	DF	SS	MS	F	P
CO_2	1	7.7E-05	7.7E-05	0.173	0.68
Nutrients	1	0.00285	0.00285	6.394	0.015
Time	5	0.0161	0.00321	7.201	<0.001
CO_2 x Nutrients	1	3.4E-07	3.4E-07	0.00077	0.978
CO_2 x Time	5	0.00102	0.0002	0.455	0.807
Nutrients x Time	5	0.0026	0.00052	1.164	0.342
CO_2 x Nutrients x Time	5	0.00042	8.4E-05	0.188	0.966
Residual	44	0.0196	0.00045		
Total	67	0.0425	0.00063		

Three-way analysis of variance of the tissue C:N for *Ulva* for June through November experiments

Source of Variation	DF	SS	MS	F	P
CO_2	1	4.052	4.052	0.303	0.585
Nutrients	1	312.069	312.069	23.321	<0.001
Time	5	1803.45	360.689	26.954	<0.001
CO_2 x Nutrients	1	1.895	1.895	0.142	0.709
CO_2 x Time	5	11.709	2.342	0.175	0.971
Nutrients x Time	5	931.409	186.282	13.921	<0.001
CO_2 x Nutrients x Time	5	25.172	5.034	0.376	0.862
Residual	44	588.79	13.382		
Total	67	3754.06	56.031		

Three-way analysis of variance of the tissue $\delta^{13}C$ for *Gracilaria* for August through November experiments

Source of Variation	DF	SS	MS	F	P
CO_2	1	755.986	755.986	166.194	<0.001
Nutrients	1	1.128	1.128	0.248	0.622
Time	3	164.397	54.799	12.047	<0.001
CO_2 x Nutrients	1	0.731	0.731	0.161	0.691
CO_2 x Time	3	123.895	41.298	9.079	<0.001
Nutrients x Time	3	5.598	1.866	0.41	0.747
CO_2 x Nutrients x Time	3	2.675	0.892	0.196	0.898
Residual	30	136.465	4.549		
Total	45	1229.61	27.325		

Three-way analysis of variance of the tissue δ^{13}C for *Ulva* for the August through November experiments

Source of Variation	DF	SS	MS	F	P
CO_2	1	2828.64	2828.64	878.483	<0.001
Nutrients	1	60.71	60.71	18.855	<0.001
Time	2	131.475	65.737	20.416	<0.001
CO_2 x Nutrients	1	0.0851	0.0851	0.0264	0.872
CO_2 x Time	2	28.94	14.47	4.494	0.022
Nutrients x Time	2	16.984	8.492	2.637	0.092
CO_2 x Nutrients x Time	2	22.594	11.297	3.508	0.046
Residual	24	77.278	3.22		
Total	35	3166.71	90.477		

One-way ANOVA of the δ^{13}C content of *Gracilaria* exposed elevated CO_2 conditions compared with the δ^{13}C signature expected from the exclusive use of CO_2 or the exclusive use of HCO_3^- (Fig 4). Tukey tests indicated each group was significantly different from each other.

Source of Variation	DF	SS	MS	F	P
Between Groups	2	2124.84	1062.42	119.986	<0.001
Residual	27	239.072	8.855		
Total	29	2363.91			

One-way ANOVA of the δ^{13}C content of *Ulva* exposed elevated CO_2 conditions compared with the δ^{13}C signature expected from the exclusive use of CO_2 or the exclusive use of HCO_3^- (Fig 4). Tukey tests indicated each group was significantly different from each other.

Source of Variation	DF	SS	MS	F	P
Between Groups	2	1210.59	605.296	47.185	<0.001
Residual	33	423.331	12.828		
Total	35	1633.92			
