# Supplementary material for: Ocean Acidification Accelerates the Growth of Two Bloom-Forming Macroalgae
Source: PLoS One. 2016 May 13;11(5):e0155152. doi: 10.1371/journal.pone.0155152 (PMC4866684; doi:10.1371/journal.pone.0155152)
Supplement: S3 Table — Values represent means ± SE. (PDF) [file pone.0155152.s003.pdf]

Supporting Information for: Ocean acidification accelerates the growth of two bloom-forming, estuarine macroalgae

Craig S. Young and Christopher J. Gobler

Supplementary Tables

S3 Tables. Tissue $\delta^{13}C$ content (‰) of dry tissue samples of *Gracilaria* and *Ulva* for August through November experiments. Values represent means ± SE.

Gracilaria

Treatment	August	Early September	Early October	November
Control	-13.32±1.29	-10.63±0.16	-13.22±0.47	-13.82±0.61
Nutrients	-12.32±0.49	-11.81±0.51	-13.19±0.44	-13.42±0.97
CO_2	-18.21±0.79	-21.09±2.30	-18.08±1.05	-27.35±2.61
CO_2/Nutrients	-15.26±2.24	-18.58±2.27	-18.71±1.40	-25.47±1.64

Ulva

Treatment	August	Early September	November
Control	-6.57±1.09	-6.70±1.16	-11.10±0.34
Nutrients	-4.64±0.83	-3.87±0.21	-8.36±0.39
CO_2	-24.15±0.73	-27.67±1.75	-26.02±0.82
CO_2/Nutrients	-19.02±1.24	-23.29±1.34	-27.46±1.32
