# Supplementary material for: Ocean Acidification Accelerates the Growth of Two Bloom-Forming Macroalgae
Source: PLoS One. 2016 May 13;11(5):e0155152. doi: 10.1371/journal.pone.0155152 (PMC4866684; doi:10.1371/journal.pone.0155152)
Supplement: S4 Table — Values represent means ± SE. (PDF) [file pone.0155152.s004.pdf]

Supporting Information for: Ocean acidification accelerates the growth of two bloom-forming, estuarine macroalgae

Craig S. Young and Christopher J. Gobler

Supplementary Tables

S4 Tables. Tissue nitrogen content (g N per g dry tissue), tissue carbon content (g N per g dry tissue), and tissue C:N of dry tissue samples of *Gracilaria* and *Ulva* for August through November experiments. Values represent means ± SE.

Gracilaria

Tissue nitrogen content

Treatment	August	Early September	Late September	Early October	Late October	November
Control	0.021±0.002	0.027±0.002	0.031±0.004	0.033±0.001	0.035±0.002	0.032±0.001
Nutrients	0.021±0.001	0.033±0.001	0.033±0.002	0.034±0.001	0.036±0.001	0.035±0.001
CO_2	0.021±0.004	0.028±0.001	0.031±0.004	0.034±0.001	0.035±0.002	0.027±0.002
CO_2/Nutrients	0.027±0.002	0.031±0.001	0.034±0.001	0.033±0.001	0.034±0.001	0.032±0.002

Tissue carbon content

Treatment	August	Early September	Late September	Early October	Late October	November
Control	0.308±0.022	0.315±0.009	0.293±0.019	0.291±0.001	0.311±0.017	0.303±0.006
Nutrients	0.225±0.015	0.321±0.015	0.263±0.008	0.298±0.009	0.317±0.015	0.278±0.008
CO_2	0.280±0.022	0.330±0.001	0.284±0.013	0.322±0.008	0.303±0.003	0.275±0.014
CO_2/Nutrients	0.298±0.014	0.316±0.017	0.292±0.009	0.302±0.011	0.310±0.005	0.278±0.016

Tissue C:N

Treatment	August	Early September	Late September	Early October	Late October	November
Control	17.2±0.3	13.6±0.6	11.1±0.8	10.2±0.1	10.5±1.0	11.1±0.2
Nutrients	12.3±0.2	11.2±0.5	9.3±0.3	10.3±0.5	10.4±0.5	9.4±0.3
CO_2	15.9±1.6	14.0±0.5	11.1±0.9	11.1±0.6	10.2±0.5	12.1±0.6
CO_2/Nutrients	12.8±0.6	12.1±0.5	10.0±0.4	10.5±0.1	10.5±0.1	10.1±0.6

Ulva

Tissue nitrogen content

Treatment	August	Early September	Late September	Early October	Late October	November
Control	0.012±0.001	0.011±0.001	0.015±0.001	0.024±0.003	0.027±0.001	0.023±0.001
Nutrients	0.026±0.001	0.018±0.003	0.018±0.001	0.024±0.003	0.025±0.001	0.023±0.001
CO_2	0.012±0.001	0.012±0.003	0.019±0.001	0.024±0.001	0.025±0.001	0.024±0.002
CO_2/Nutrients	0.027±0.002	0.018±0.001	0.019±0.003	0.025±0.001	0.024±0.001	0.022±0.001

Tissue carbon content

Treatment	August	Early September	Late September	Early October	Late October	November
Control	0.302±0.003	0.335±0.009	0.267±0.003	0.295±0.019	0.301±0.019	0.293±0.011
Nutrients	0.335±0.013	0.322±0.007	0.282±0.018	0.309±0.013	0.320±0.003	0.306±0.010
CO_2	0.295±0.005	0.327±0.009	0.268±0.034	0.306±0.011	0.286±0.011	0.300±0.010
CO_2/Nutrients	0.313±0.017	0.321±0.016	0.282±0.011	0.317±0.013	0.322±0.004	0.304±0.007

Tissue C:N

Treatment	August	Early September	Late September	Early October	Late October	November
Control	29.3±2.8	36.7±2.0	21.5±1.9	14.5±0.8	12.9±0.3	14.7±0.3
Nutrients	15.3±0.6	21.9±2.9	18.0±0.7	15.3±1.2	15.2±0.4	15.8±0.5
CO_2	29.8±3.2	35.1±6.3	16.5±1.1	14.7±0.5	13.5±0.8	14.8±1.4
CO_2/Nutrients	13.8±0.5	21.2±1.7	19.0±3.7	15.1±0.5	15.6±0.1	15.8±0.4
